# Supplementary material for: Content-rich biological network constructed by mining PubMed abstracts
Source: BMC Bioinformatics. 2004 Oct 8;5:147. doi: 10.1186/1471-2105-5-147 (PMC528731; doi:10.1186/1471-2105-5-147)
Supplement: Additional File 2 — The original results of the above study (non-essential files are deleted to keep the file size under the limit set by BMC bioinformatics). [file 1471-2105-5-147-S2.bz2 › chilibotAdditionalFile2/dip05/720ID11777003E18057/html/RPOE_RSEB.html]

 


 **RPOE** and **RSEB** 
  
Found 3 abstracts in PubMed, retrieved 3.  
 

 What does Google say? 
 PDF only 
| .edu only 

---

**Interactive relationship** (e.g. stimulation, inhibition, etc)

**Non-interactive relationship** (e.g. studied together, co-existance, homology, etc.)

- An insertion in  **rseB** , the third gene in the  **rpoE**  rseABC gene cluster at 57 min, prevented HMP synthesis in a purF mutant.  Ref: 9335303 J Bacteriol, 1997
- Modulation of the Escherichia coli sigmaE  **RpoE**  heat shock transcription factor activity by the RseA,  **RseB**  and RseC proteins.  Ref: 9159522 Mol Microbiol, 1997
- Nucleotide sequence analysis of this product revealed that it spanned the 3 and 5 ends of  **rpoE**  and  **rseB** , respectively, which form part of the extracytoplasmic stress operon that encodes and regulates expression of alternate sigma factor sigma E final sigma E.  Ref: 11796603 Infect Immun, 2002
